# Snuff use: Motivations, tips to quit, and readiness to quit in a South African township

**DOI:** 10.4102/jcmsa.v2i1.70

**Published:** 2024-09-03

**Authors:** Tombo Bongongo, Jeewa Yusuf, Indiran Govender, Doudou K. Nzaumvila, Sunday Okeke, Carien Steyn

**Affiliations:** 1 Medicine Academic Department, School of Medicine, Sefako Makgatho Health Sciences University, Pretoria, South Africa; 2 Tshwane Health District, Sefako Makgatho Health Sciences University, Pretoria, South Africa; 3 Department of Family Medicine and Primary Health Care, School of Medicine, Sefako Makgatho Health Sciences University, Pretoria South Africa

**Keywords:** snuff, motivations, tips for quitting, readiness to quit, Pretoria, South Africa

## Abstract

**Background:**

Regardless of how tobacco products are consumed, they increase the risk of morbidity and mortality for users. This study aims to determine the motivations for using snuff, assess awareness of tips to quit, and determine readiness to quit snuff use in patients at a clinic in a Pretoria township, in South Africa.

**Methods:**

A descriptive cross-sectional survey using a piloted, structured, and self-administered questionnaire was given to patients at Ramotse clinic.

**Results:**

The mean age of the 402 participants was 49.0 years, with a range of 18–104 years. The majority (83.8%) of the participants were females. Of the participants, 26.6% were 60 years of age or older, 59.7% were unemployed, 57.5% were married, and 41.5% had finished primary school. Socio-cultural practices and behaviour were mentioned by 38.1% as the main reason for snuff usage, while 77.2% were uninformed about tips for quitting snuff, and 63.25% were unwilling to give up snuff.

**Conclusion:**

The study found that unemployed, married, mostly female, 40 years of age or older, and had a basic education match with the profile of participants. Socio-cultural practices and behaviour and health benefits influenced the habit. Most participants were unaware of tips to quit habits and unprepared to do so.

**Contribution:**

Healthcare professionals need to be aware of the health hazards that their patients may face, make sure they are equipped to address them and offer community-saving tips for improvement.

## Introduction

Smokeless tobacco (ST) use is the practice of using incombustible tobacco products without smoking.^1,2^ Available forms of ST are snuff, snus, heated tobacco products and chewing, oral or spitting tobacco,^1^ and all of them contain nicotine.^2^ The use of the snuff by quite a few patients attending one of the Pretoria day clinics has impacted the minds of a few medical doctors working in primary care in Pretoria to initiate this study.

The use of snuff was first recorded among American Indians to prevent fatigue and relieve hunger, as well as for ritual and ceremonial practices.^2^ In ancient China, it was believed that snuff could induce sweating, cure toothache, and relieve constipation.^2^ Snuff use became popular in Europe in the 16th–18th centuries, when it was often used by royalty.^2^

Tobacco use is responsible for approximately 15% of all global deaths – nearly nine million deaths annually.^3^ A recent systematic review on the health outcomes of ST use revealed overwhelming evidence that ST is associated with a variety of adverse health outcomes, especially in Africa, Asia and the Middle East.^4^ These outcomes include increased mortality rates, risk for a higher number of malignancies (such as oesophageal cancer and oral cancer),^2^ coronary heart disease, stroke, hypertension and dyslipidaemia,^3,4^ as well as dental and periodontal disease and adverse pregnancy outcomes.^2^ In addition to these adverse effects, repeated tobacco use can lead to nicotine dependence,^5^ which in turn sustains the tobacco use.^5^ Consumers rely on tobacco to modulate mood and to give a sense of arousal,^5^ which is achieved by the release of dopamine and other neurotransmitters in the brain through the action of the nicotine on cholinergic neurotransmitters.^5^ As with other addictions, tobacco addiction or dependence is a disorder that requires not only more than acute but also follow-up care because of the vulnerability of users to relapse.^5^ A decreased nicotine level in the brain that results from discontinuation of tobacco use can lead to tobacco cravings that may maintain the urge to use the substance.^5^ This process is because of lower levels of dopamine being released in the brain. Dopamine is a neurotransmitter that leads to a temporary sensation of well-being; use of ST and smoking tobacco can trigger the release of dopamine within 20 seconds of ingestion.^5^

To quit using any form of tobacco is challenging.^6^ In adults who smoke tobacco, there is a 50% lifetime chance of quitting and 42% of consumers of ST will be unable to quit.^6^ All forms of smokeless tobacco use, including snuff, have been researched in the United States (US) to help individuals give it up. Behavioural assistance and medicine were the two main strategies.^7^ With medication approach, numerous studies employed different kinds of nicotine replacement therapy (two patches, five gum and five lozenges), yet the results showed that nicotine lozenges could aid in patients’ cessation; however, the quality of the evidence was poor, necessitating further investigation. The behavioural assistance included short instruction, self-help materials, phone support, online access and feature combinations. The results were wildly inconsistent, with some trials providing strong proof of benefit and others yielding no effect at all.^7^

In order to better support those who use snuff and visit the Ramotse clinic in Pretoria, this study aims to determine the motivations behind the use of ST, especially snuff, as well as their understanding of various cessation techniques and their readiness to stop using it.

## Methods

### Study design

A descriptive cross-sectional survey using a piloted, structured and self-administered questionnaire.

### Study setting

The study was carried out at Ramotse clinic, in the suburb called Ramotse, in Pretoria, in the northern Gauteng province of South Africa. It is situated along the national road referred to as N1. This is a day clinic (works from 6:00 to 16:00) with a daily headcount of 300 and a catchment population of around 20 000. It provides services such as chronic care (hypertension, diabetes mellitus, human immunodeficiency virus-acquired immunodeficiency syndrome [HIV-AIDS], and so on), ante-natal and post-natal care, mental health, psychology, dental care, social worker, dietician and acute illness care.

### Population, sampling and sample size

The sample size was determined using Ramotse’s population, which was roughly 15 760 according to the 2011 census.^8^ The required sample size for such a population was determined to be 376 (*n* = 376) using the net sample size formula, also known as the Raosoft sample size calculator,^9^ with a 95% confidence level and a 5% margin of error. The final sample size of 402 (*n* = 402) was determined by tolerating oversampling during the data collection phase because of participants’ willingness to be part of the survey. This was a convenience sampling because only those who were available and willing to offer the studied information were selected.

### Data collection

After having been informed about the study’s objectives and aim by the first research assistant (RA) while in the waiting area of the clinic, willing participants met with the second RA once they were done with the consultation and pharmacy. They were given the informed consent form and the questionnaire. Those who were unable to complete the two documents received assistance from the second RA. Only participants who used snuff and were at least 18 years old were recruited.

### Data collection tool

The questionnaire was created by the main author in accordance with the study’s aim and objectives. The New Eersterus clinic was chosen for the pilot study because it is a day clinic in a distant location (30 km), comparable to the Ramotse clinic and most of their patients have the same socio-cultural traits such as language (Tswana and English). To confirm the tool’s validity, participants were exposed to the questionnaire for the first time in the New Eesterus clinic; thereafter, they provided feedback to ensure that it appropriately assessed the aim and objectives of the survey (face validity).^10^ The New Eesterus clinic nurses administered the identical questionnaire to three patients with chronic diseases on the pilot day to ensure test-retest reliability. These patients were well-known as snuff users at the clinic. These three recruited participants were told to return in two weeks, and their responses to the questionnaire on day one mirrored those on day fourteen.^10^

The questionnaire was divided into four sections: (1) socio-demographics (age, gender, marital status, educational level, and employment status), (2) motivation for using snuff (peer pressure, socio-cultural or behaviour, addiction to tobacco products, health benefits such as treat headaches, for fun, cheaper that cigarette, other reasons not mentioned above), (3) awareness of tips for quitting snuff (pick a quit date to stop snuff use, understand nicotine withdrawal signs, learn how to handle triggers and craving, learn to substitute or replace snuff with nicotine replacement therapy, try medications or tablets that help to stop snuff use, need support groups), and (4) readiness to cease the snuff taking (yes, no, not sure).

The questionnaire used two of the languages (English and Tswana) most spoken in the area. Interpretation of the questionnaire in Tswana was completed by two RAs who are confident in reading and writing the two languages. A Tswana teacher at Ramotse School did the back translation. This helped to ensure that the meaning in both languages was the same. Data collection spanned nearly 7 months, from June 2021 to January 2022.

### Data analysis

Data were captured in a Microsoft Excel spreadsheet and then imported into STATA version 14 for coding and analysis. Descriptive statistics were employed to describe the demographics of the participants, their reasons for using snuff, their awareness of cessation strategies, and their willingness to quit snuff. Continuous variables were represented by their mean and standard deviation, whereas categorical variables were described using frequencies and percentages.

### Participants’ characteristics

A total of 402 patients participated in this study (Table 1). Their mean age was 49.0 years (standard deviation [s.d.] ±19.1), and range of 18–104 years.

**TABLE 1:** Demographic characteristics of the participants.

Variables	Frequency (*n*)	%
**Age (in years)**		
< 20	7	1.7
20–29	45	11.2
30–39	98	24.4
40–49	103	25.6
50–59	42	10.4
60+	107	26.6
Unspecified	1	0.2
**Gender**		
Male	63	15.7
Female	337	83.8
Unspecified	2	0.5
**Employment status**		
Employed	160	39.8
Unemployed	240	59.7
Unspecified	2	0.5
**Marital status**		
Single	73	18.2
Married	231	57.5
Divorced	58	14.4
Widow	37	9.2
Unspecified	3	0.7
**Level of education**		
None	41	10.2
Primary	167	41.5
Secondary	150	37.3
Tertiary	26	6.5
Unspecified	18	4.5

Of the 402 participants, 38.1% stated that socio-cultural practices and behaviour was the primary reason behind the use of ST products (snuff), whereas 22.1% stated that snuff offers numerous health benefits, as shown in Table 2.

**TABLE 2:** Reasons given for using snuff.

Variables	Frequency (*n*)	%
1. Peer pressure	45	11.2
2. Socio-cultural practices/behaviour	153	38.1
3. Addiction to tobacco product	47	11.7
4. Snuff has many health benefits such as in the headaches, toothache, etc.	89	22.1
5. For fun	16	4.0
6. Snuff is cheaper than cigarette	46	11.4
7. Others that are not above-mentioned	6	1.5

**Total**	**402**	**100.0**

Table 3 shows that a high majority of the 400 participants who completed the table on awareness of quitting snuff were unaware or unsure of the tips.

**TABLE 3:** Tips for quitting the behaviour (snuff).

Tips for quitting snuff use	*N*	Yes		No		Not sure	
		*n*	%	*n*	%	*n*	%
1. Pick a quit date to stop snuff use.	400	56	14	227	57	117	29
2. Understand nicotine withdrawal signs.	400	92	23	201	50	107	27
3. Learn how to handle triggers and craving.	400	77	19	174	44	149	37
4. Learn to substitute or replace snuff with nicotine replacement therapy.	400	55	14	175	44	170	42
5. Try medications that help to stop snuff use.	400	62	16	189	47	149	37
6. Need support groups.	400	83	21	173	43	144	36

**FIGURE 1:**
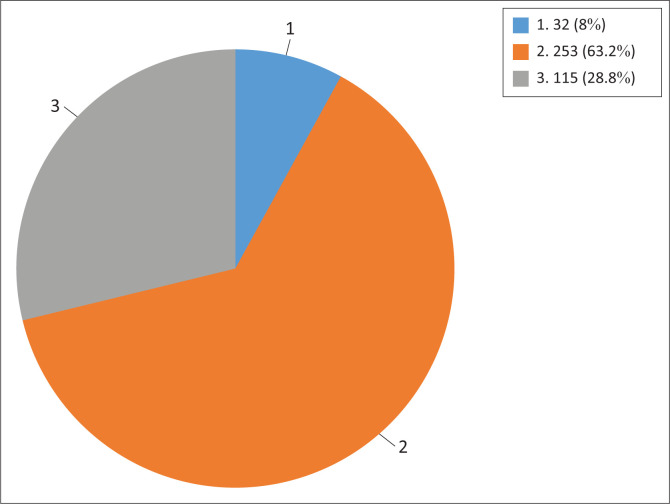
Those who were ready to quit taking snuff.

Only 400 people responded to the questionnaire’s portion about their readiness to cease using snuff. The results indicated that the majority (63.25%) are unwilling to give up snuff (Figure 1).

## Discussion

Data on snuff usage in a semi-urban Pretoria region can be grouped in four points: (1) The sociodemographic data of the participants revealed that most of them were unemployed, married, mostly female, 40 years of age or older, and had only a basic education; (2) Motivations supporting the behaviour: when asked about the reasons that influenced their behaviour, most of them cited socio-cultural practices and behaviours as well as the health advantages that the goods they utilised offered; (3) In terms of awareness on tips that can help them quit the habit, most of them were unaware, and (4) when it came to ready to stop the behaviour, most of them were not ready.

From the sociodemographic viewpoint, there are some parallels to Nigerian research published in 2021^11^ that looked at the prevalence, patterns, and correlates of ST use.^11^ The most common kind of ST used in Nigeria was snuff by nose. And, while this survey did not compare the usage of the various varieties of ST, one of the studies on ST conducted in Ramotse has already indicated that snuff was the most common variety.^1^ In terms of education, over half of the Nigerian sample had no formal education or had only completed basic school.^11^ This is similar to the Ramotse study, in which more persons did not have more than elementary schooling. Contrary to the findings of the Ramotse survey, which revealed that females used snuff more frequently, research in Africa has shown that approximately 3% of men and 1% of women used ST on average between 2012 and 2022.^12^ This is without any information on motives or awareness of tips to quit.

In 2019, the Truth Initiative produced a study on the prevalence of ‘every day’ or ‘someday’ ST usage by age group. When the Ramotes survey indicated greater usage among those aged 40 years and up, the Truth Initiative emphasised 3.7% for those aged 45 years and over, and the total computed proportion for those under 45 years was 5.4%. According to the research, these data change over time and between regions of the world.^13^ This statement accommodates whatever finding may occur elsewhere, including Ramotse.

Regarding the motives attached to the usage of snuff, this study found that socio-cultural practices or behaviours, followed by the health advantages provided by snuff, were the primary motivators for use. In Nigeria, research performed in 2021 found that socio-cultural structure was the primary factor for ST consumption. While exploring the purpose, subthemes such as ‘culture and living conditions’, ‘laws’, ‘family and peer connections’, ‘beliefs connected to psychological’ and ‘beliefs related to physical effects’, ‘beliefs’ emerged.^14^ Despite being part of a quantitative research, these socio-cultural practices and behaviours in Ramotse were more closely related to the participants’ culture, religion or spirituality, behaviour, and living situations.

In the US, after studying all ST usages, including snuff, using two approaches (medication and behaviour support), the evidence that nicotine lozenges may help patients quit the habit was low, whereas behaviour support in its variety of components demonstrated evidence of benefit, with others having no impact.^7^ Although the Ramotse survey was not experimental, some strategies such as nicotine replacement treatment, pharmaceutical concerns, and support were mentioned in the questionnaire. As was the case in America, most participants had not been exposed to these and were unaware of these strategies. As a result, further work is needed in both contexts to address the conduct. This revelation, in Ramotse, explains members’ desire to continue snuffing.

### Study’s limitations and strengths

The type of research performed restricts participants to the limited alternatives offered to them. The study’s findings, which were conducted in one of Pretoria’s six areas, cannot be generalised. This study has provided insight into the conduct in the research region.

## Conclusion

The study found that unemployed, married, mostly female, 40 years of age or older, and had a basic education match with the profile of participants. Socio-cultural practices and behaviours and health benefits influenced the habit. Most participants were unaware of tips to quit habits and unprepared to do so.
